# Use of Sodium-Glucose Cotransporter 2 (SGLT2) Inhibitors in Heart Failure Without Diabetes

**DOI:** 10.7759/cureus.104397

**Published:** 2026-02-27

**Authors:** Jeilyn Jiron Vindas, Maynor Jose Lopez Mendoza, María Jennifer Valle Mena, Maria Antonieta Salazar Estrada, Asdrubal Ulloa, Nicolle Contreras Figueroa

**Affiliations:** 1 Obstetrics and Gynecology, Hospital de las Mujeres Dr. Adolfo Carit Eva (HOMACE), San José, CRI; 2 Anesthesiology and Perioperative Medicine, Hospital de las Mujeres Dr. Adolfo Carit Eva (CARITEVA), San José, CRI; 3 General Medicine, Área de Salud Upala, Alajuela, CRI; 4 Emergency Medicine, Hospital Los Chiles CR, Los Chiles, CRI; 5 Gynecologic Oncology, Hospital de las Mujeres Dr. Adolfo Carit Eva (HOMACE), San José, CRI; 6 General Medicine, Caja Costarricense de Seguro Social (CCSS), San José, CRI

**Keywords:** clinical outcomes, guideline-directed therapy, heart failure, ketone bodies, myocardial energy metabolism, sglt2 inhibitors

## Abstract

Sodium-glucose cotransporter 2 (SGLT2) inhibitors have become foundational therapies in the management of heart failure, extending beyond their original indication as glucose-lowering agents. Experimental and clinical evidence indicates that their cardioprotective effects are largely independent of glycemic control and are mediated through integrated hemodynamic, metabolic, and anti-inflammatory mechanisms. At the myocardial level, SGLT2 inhibitors promote a shift in substrate utilization toward fatty acids and ketone bodies, improving mitochondrial efficiency and cellular energy balance. These effects are accompanied by reductions in oxidative stress, inflammation, and maladaptive remodeling, as well as favorable vascular and cardiorenal interactions. Large randomized controlled trials have consistently demonstrated significant reductions in heart failure hospitalization across patients with and without diabetes. DAPA-HF and EMPEROR-Reduced established efficacy in heart failure with reduced ejection fraction, while EMPEROR-Preserved and DELIVER extended these benefits to patients with mildly reduced and preserved ejection fractions. Although reductions in cardiovascular mortality are most robust in reduced ejection fraction, improvements in morbidity and health-related quality of life have been observed across phenotypes. With a generally favorable safety profile in nondiabetic populations and strong Class I guideline recommendations in heart failure with reduced ejection fraction, SGLT2 inhibitors are now recognized as one of the four core pillars of guideline-directed medical therapy. Their widespread implementation represents a major advance in disease-modifying treatment, though optimization of real-world uptake remains an ongoing clinical priority.

## Introduction and background

Heart failure is a leading cause of morbidity and mortality worldwide and affects millions of individuals, including a substantial proportion of patients without diabetes. The significant prevalence of heart failure in nondiabetic populations highlights the need for therapeutic strategies capable of addressing cardiovascular risk independently of glycemic status [1]. Importantly, patients with heart failure who do not have diabetes often experience clinical outcomes comparable to those observed in diabetic populations, including high rates of hospitalization, disease progression, and mortality [2].

The epidemiologic relationship between diabetes and heart failure is well established. According to a consensus report from the American Diabetes Association, up to 22% of individuals with diabetes may develop heart failure, even in the absence of other major cardiovascular conditions [1]. Data from the Atherosclerosis Risk in Communities study demonstrate that the risk of incident heart failure increases with diabetes duration, with individuals living with diabetes for 15 years or more exhibiting a hazard ratio of 2.82 compared with those without diabetes [2]. Similarly, findings from the UK Biobank cohort indicate that individuals with diabetes have nearly double the risk of developing heart failure (HR: 1.9) relative to nondiabetic counterparts [3]. However, enrollment data from landmark trials such as EMPEROR-Reduced and DAPA-HF indicate that approximately 55-60% of patients with heart failure did not have diabetes, highlighting that nondiabetic individuals represent a substantial, and often majority, proportion of the heart failure population [4,5].

In parallel with this clinical burden, heart failure pharmacotherapy has evolved beyond therapies focused exclusively on hemodynamic modulation. Sodium-glucose cotransporter 2 (SGLT2) inhibitors, originally developed as glucose-lowering agents, have emerged as integral components of heart failure management due to robust cardiovascular outcome data. Large-scale trials have demonstrated significant reductions in heart failure hospitalization and cardiovascular mortality across broad patient populations, with benefits observed irrespective of diabetes status [3,4]. In particular, EMPEROR-Reduced and DAPA-HF showed that empagliflozin and dapagliflozin improve outcomes in heart failure with reduced ejection fraction, with consistent effects in both diabetic and nondiabetic subgroups [5].

The rationale for focusing specifically on nondiabetic heart failure populations is therefore supported by both epidemiologic data and clinical trial evidence. Beyond glucose lowering, SGLT2 inhibitors exert hemodynamic, metabolic, and anti-inflammatory effects that contribute to improved cardiovascular outcomes [6,7]. Consistent findings across multiple trials demonstrate reductions in heart failure hospitalization and composite cardiovascular endpoints in patients without diabetes [1,8], and emerging evidence in preserved and mildly reduced ejection fraction further supports their role as disease-modifying therapies across the spectrum of heart failure phenotypes [9].

The objective of this article is to critically review and synthesize the current evidence on the use of SGLT2 inhibitors in the management of heart failure in patients without diabetes, with particular emphasis on their pathophysiological rationale, clinical efficacy across different heart failure phenotypes, safety profile, and impact on cardiovascular and renal outcomes, in order to clarify their role within contemporary guideline-directed heart failure therapy independent of glycemic status.

## Review

Methods

This manuscript was conducted as a structured narrative review. A literature search was performed using established bibliographic databases, including PubMed, ScienceDirect, and the Cochrane Library. Searches were conducted between January 2024 and February 2026. High-impact cardiovascular journals were additionally screened when necessary to ensure inclusion of landmark randomized trials and major guideline documents.

The search strategy combined keywords and controlled vocabulary terms related to “SGLT2 inhibitors”, “heart failure”, “reduced ejection fraction”, “preserved ejection fraction”, “nondiabetic”, “clinical outcomes”, “myocardial metabolism”, “renal outcomes”, and “guideline-directed therapy”. Searches were limited to articles published in English or Spanish. Emphasis was placed on contemporary literature (primarily 2019-2025), while seminal trials preceding this period were included when directly relevant to foundational evidence.

The initial search identified approximately 186 records. After screening titles and abstracts for relevance and removing clearly non-pertinent publications (e.g., basic science-only reports, non-cardiovascular indications, and case reports), 74 articles were reviewed in full text. Ultimately, 31 publications were selected for inclusion based on clinical relevance, methodological robustness, and contribution to the central objective of evaluating SGLT2 inhibitors in heart failure independent of diabetes status.

Priority was given to large randomized controlled trials, pooled analyses, high-quality meta-analyses, registry-based studies addressing real-world implementation, and major international guideline statements. Observational studies were included selectively when they contributed meaningful data regarding safety, prescribing patterns, or nondiabetic subgroups. Preclinical studies were cited only to contextualize mechanistic concepts.

Given the narrative design of this review, no formal systematic review framework (e.g., Preferred Reporting Items for Systematic reviews and Meta-Analyses (PRISMA) flow diagram), duplicate independent screening process, or quantitative risk-of-bias synthesis was performed. The objective was to provide a clinically oriented synthesis of current evidence rather than a reproducible meta-analytic evaluation.

Artificial intelligence tools were used solely to assist with structural organization and linguistic refinement of the manuscript. All literature identification, selection decisions, data interpretation, and scientific judgment were performed exclusively by the authors.

Overview of SGLT2 inhibitors

SGLT2 inhibitors exert cardioprotective effects that are largely independent of their glucose-lowering properties through multiple interrelated mechanisms. Heart failure is characterized by myocardial metabolic inflexibility, in which the failing myocardium exhibits impaired substrate switching, dysfunctional mitochondrial calcium handling, reduced efficiency of the electron transport chain, and increased generation of reactive oxygen species. These alterations contribute to diminished adenosine triphosphate production, impaired oxidative phosphorylation coupling, and reduced contractile efficiency [7,10].

**Figure 1:**
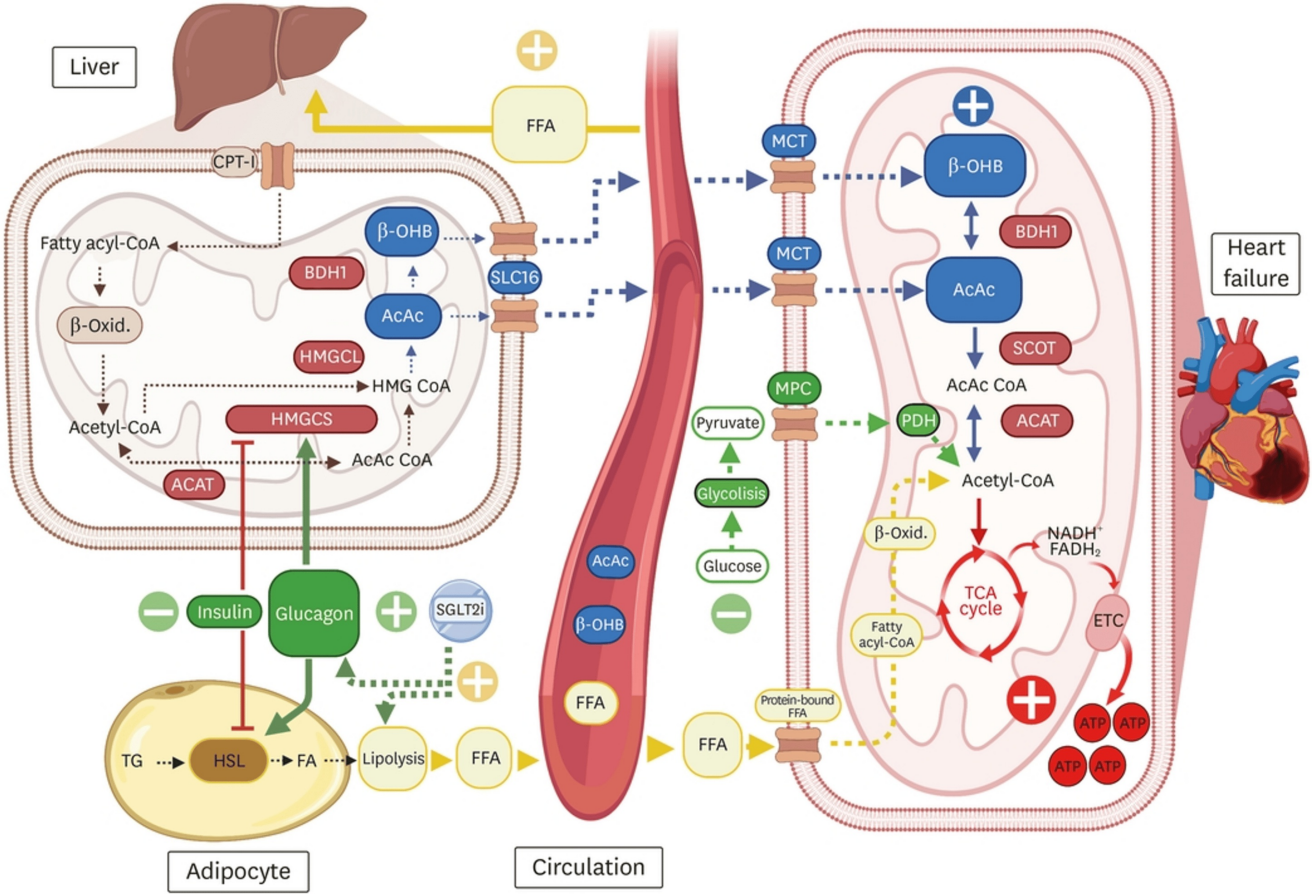
Metabolic mechanisms underlying the cardioprotective effects of SGLT2 inhibitors: ketone body production and myocardial energy utilization SGLT2 inhibitors promote enhanced lipolysis and hepatic ketogenesis, increasing circulating free fatty acids and ketone bodies, which are subsequently utilized by the failing myocardium to improve mitochondrial ATP production. These metabolic adaptations may enhance oxidative phosphorylation efficiency and support myocardial energetic balance in heart failure. “+” indicates stimulation or upregulation, while “-” indicates inhibition or downregulation. Dashed arrows represent transport processes between compartments, and solid arrows indicate metabolic pathways. ACAT, acetyl-CoA acetyltransferase; AcAc, acetoacetate; ATP, adenosine triphosphate; β-OHB, beta-hydroxybutyrate; β-Oxid., beta-oxidation; BDH1, β-hydroxybutyrate dehydrogenase 1; CPT-1, carnitine palmitoyltransferase 1; ETC, electron transport chain; FA, fatty acids; FFA, free fatty acids; FADH₂, reduced flavin adenine dinucleotide; HMGCL, 3-hydroxy-3-methylglutaryl-CoA lyase; HMGCS, 3-hydroxy-3-methylglutaryl-CoA synthase; HSL, hormone-sensitive lipase; MCT, monocarboxylate transporter; MPC, mitochondrial pyruvate carrier; NADH, reduced nicotinamide adenine dinucleotide; PDH, pyruvate dehydrogenase; SCOT, succinyl-CoA:3-ketoacid CoA transferase; SGLT2i, sodium-glucose cotransporter 2 inhibitor; SLC16, solute carrier family 16 transporter; TCA cycle, tricarboxylic acid cycle; TG, triglycerides This figure is reproduced from Saucedo-Orozco et al. (2022) [11]; Creative Commons Attribution-NonCommercial (CC BY-NC) 4.0.

SGLT2 inhibitors modulate myocardial energy metabolism by promoting a shift in substrate utilization from carbohydrates toward fatty acids and ketone bodies, fuels that may be metabolically more efficient under conditions of energetic stress. This shift enhances mitochondrial efficiency and improves cardiac energetic balance. In parallel, these agents reduce oxidative stress and attenuate inflammatory pathways, both of which play central roles in the progression of heart failure and myocardial dysfunction [10]. These metabolic adaptations, particularly enhanced ketone body utilization, are illustrated in Figure 1.

Beyond their direct myocardial effects, SGLT2 inhibitors also exert favorable actions on the vascular system, including reductions in vascular stiffness and calcification, as well as improvements in endothelial function. These vascular benefits are mediated through endothelial cell protection and modulation of inflammatory signaling, ultimately enhancing vasodilation and contributing to overall cardiovascular protection [6].

Among the approved agents within this drug class, empagliflozin and dapagliflozin have demonstrated the most robust evidence for cardiovascular benefit. These agents are associated with modest but clinically meaningful reductions in blood pressure and exhibit anti-inflammatory properties that further support their cardioprotective profile [12,13]. Based on consistent findings from large randomized clinical trials, empagliflozin and dapagliflozin have been incorporated into contemporary heart failure management guidelines, reflecting their ability to significantly reduce hospitalizations for heart failure irrespective of diabetes status [14].

Although the glucose-lowering effects of SGLT2 inhibitors are primarily mediated through enhanced renal glucose excretion, their cardiovascular benefits arise from distinct, glucose-independent mechanisms. These off-target effects include improvements in cellular energy metabolism, reductions in oxidative stress, and modulation of inflammatory and fibrotic pathways, which collectively contribute to slowing disease progression. As a result, the cardioprotective actions of these agents extend beyond glycemic control and encompass reductions in heart failure progression and structural cardiovascular deterioration, supporting their therapeutic value in both diabetic and nondiabetic populations [14,15].

Evidence from clinical trials

Evidence from major randomized controlled trials has established SGLT2 inhibitors as effective therapies across the spectrum of heart failure, including patients without diabetes. In DAPA-HF, dapagliflozin reduced the composite of cardiovascular death or worsening heart failure (HR: 0.74; 95% CI: 0.65-0.85; p < 0.001). Similarly, in EMPEROR-Reduced, empagliflozin reduced the composite of cardiovascular death or heart failure hospitalization (HR: 0.75; 95% CI: 0.65-0.86; p < 0.001). In both trials, treatment effects were consistent in patients with and without diabetes, with no significant interaction by diabetic status, supporting the glycemic-independent benefit of these agents [3,16].

The EMPEROR-Preserved and DELIVER trials extended these findings to heart failure with preserved or mildly reduced ejection fraction. In EMPEROR-Preserved, empagliflozin reduced the primary composite endpoint (HR: 0.79; 95% CI: 0.69-0.90; p < 0.001), largely driven by a reduction in heart failure hospitalizations. DELIVER demonstrated a similar benefit with dapagliflozin (HR: 0.82; 95% CI: 0.73-0.92; p < 0.001). In both studies, subgroup analyses showed consistent effects irrespective of diabetes status, with no statistically significant interaction, reinforcing the applicability of SGLT2 inhibitors in nondiabetic populations [8,17].

Subgroup analyses specifically evaluating patients without diabetes demonstrated that reductions in heart failure hospitalization were the primary contributors to clinical benefit. Although absolute event rates varied, the relative risk reductions remained comparable between diabetic and nondiabetic participants. These findings indicate statistical consistency rather than mere directional similarity, as formal interaction testing did not demonstrate heterogeneity of treatment effect [1,16,18].

Regarding cardiovascular mortality, benefits have been more pronounced in heart failure with reduced ejection fraction than in preserved ejection fraction. While mortality reductions in nondiabetic subgroups did not consistently reach statistical significance in every individual trial, pooled analyses suggest a coherent reduction in composite outcomes driven predominantly by decreased heart failure events. In addition, SGLT2 inhibitor therapy has been associated with clinically meaningful improvements in health-related quality of life, as measured by the Kansas City Cardiomyopathy Questionnaire, across heart failure phenotypes and independent of diabetes status [4,19].

Efficacy across heart failure phenotypes

SLGT2 inhibitors have become central to contemporary heart failure management across ejection fraction phenotypes, reflecting a progressively broader evidence base and corresponding guideline endorsements. In heart failure with reduced ejection fraction, these agents have achieved a Class I recommendation due to their established efficacy in reducing hospitalizations and cardiovascular deaths, consolidating their role as a foundational component of guideline-directed medical therapy [20-22]. Within this phenotype, dapagliflozin has been highlighted for demonstrating significant reductions in hospitalization risk, cardiovascular death, and all-cause mortality, reinforcing the clinical relevance of SGLT2 inhibition in high-risk heart failure populations [23,24].

The expansion of SGLT2 inhibitor use into heart failure with mildly reduced ejection fraction has been supported by accumulating trial data and meta-analytic evidence. In this context, these agents carry a Class IIa recommendation, reflecting evidence that they reduce the risk of hospitalization for heart failure and contribute to improvements in quality of life [20,21,24]. Meta-analyses further support these observations by reporting consistent reductions in the primary composite endpoint of first heart failure hospitalization or cardiovascular death among patients with mildly reduced ejection fraction, strengthening the rationale for their use in this intermediate phenotype [25].

Similarly, in heart failure with preserved ejection fraction, SGLT2 inhibitors have demonstrated clinically meaningful benefits, particularly in terms of improving cardiac function and patient-reported quality of life, although reductions in cardiovascular death appear less pronounced than those reported in heart failure with reduced ejection fraction. Within this phenotype, sotagliflozin has been reported to show superiority in reducing hospitalizations and cardiovascular death compared with other SGLT2 inhibitors, suggesting potential heterogeneity within the class that may be relevant in certain patient subgroups [23,24,26].

Across these phenotypes, the therapeutic effects of SGLT2 inhibitors appear consistent regardless of diabetic status, supporting their value as disease-modifying agents for a broad range of heart failure patients and reinforcing the concept that their principal benefits are mediated through mechanisms independent of glucose lowering [20,21,26].

Safety and tolerability in nondiabetic patients

The safety profile of SGLT2 inhibitors in nondiabetic patients with heart failure has been extensively evaluated and is generally favorable. Available evidence indicates that these agents are associated with a reduction in serious adverse events and a lower incidence of acute kidney injury in heart failure populations, although the certainty of evidence varies across analyses. Meta-analytic data suggest reductions in all-cause mortality and serious adverse events, particularly in patients with reduced ejection fraction. However, these benefits are accompanied by an increased incidence of genitourinary infections compared with placebo [27,28].

From a metabolic safety perspective, SGLT2 inhibitors do not significantly increase the risk of hypoglycemia in nondiabetic patients, consistent with their insulin-independent mechanism of action [24]. Likewise, the incidence of diabetic ketoacidosis in nondiabetic heart failure populations has been low and not significantly different from placebo in major trials [3].

Hemodynamic effects related to volume status are clinically relevant. SGLT2 inhibitors may contribute to volume depletion and symptomatic hypotension, particularly in patients receiving concomitant diuretics or with baseline low blood pressure [25]. Despite their natriuretic effects, these agents have not been associated with an increased risk of acute kidney injury in large randomized trials and may attenuate long-term decline in estimated glomerular filtration rate [27,29]. When combined with loop diuretics, SGLT2 inhibitors can improve diuretic efficiency without a corresponding increase in renal adverse events, supporting their integration into multidrug volume management strategies [3].

Genitourinary infections, particularly genital mycotic infections, represent the most consistently reported adverse events. Meta-analyses have reported an increased relative risk compared with placebo (risk ratio: approximately 3.56), although most cases are mild to moderate and respond to standard antifungal therapy [29]. In clinical practice, uncomplicated infections typically do not require permanent discontinuation. Temporary interruption may be considered in cases of severe, recurrent, or systemic infection, based on individualized clinical assessment. Safety considerations should be weighed against the demonstrated reductions in heart failure hospitalization and composite cardiovascular outcomes observed in randomized trials [30].

Practical considerations for clinical use

Appropriate patient selection is essential to optimize the clinical benefits of SGLT2 inhibitors in heart failure. Evidence supports their efficacy across a broad range of phenotypes, including reduced, mildly reduced, and preserved ejection fraction, with benefits observed across varying levels of left ventricular systolic function and renal function [20,21,26]. Trials such as DAPA-HF and EMPEROR-Reduced demonstrated significant cardiovascular benefit in patients without diabetes, confirming that therapeutic effects extend beyond glycemic control [5]. Accordingly, current guidelines recommend SGLT2 inhibitors for most patients with heart failure in the absence of contraindications [3,20,21].

SGLT2 inhibitors can generally be initiated at different stages of heart failure management, including during hospitalization for acute decompensation, as supported by the EMPULSE trial. Standard dosing consists of a fixed 10 mg once-daily regimen without the need for titration. Initiation may be deferred or undertaken with closer monitoring in patients with significant volume depletion, symptomatic hypotension, severe acute illness, or active serious genitourinary infection [20].

In routine practice, SGLT2 inhibitors are well integrated into guideline-directed medical therapy. They can be combined with loop diuretics, renin-angiotensin system inhibitors, beta-blockers, and mineralocorticoid receptor antagonists without an increased risk of renal injury in major trials. Their natriuretic effects may enhance diuretic efficiency, although volume status should be carefully assessed, particularly in patients receiving high-dose diuretics [3].

Ongoing monitoring after initiation includes evaluation of renal function, blood pressure, and volume status, especially during the early treatment phase. Clinical follow-up should also assess symptom burden and health-related quality of life, as improvements are often detectable within weeks of therapy initiation [27].

Current guideline recommendations

SGLT2 inhibitors occupy a central position in contemporary international heart failure guidelines, reflecting the strength and consistency of outcome data from randomized clinical trials. The 2022 AHA/ACC/HFSA guideline and the 2021 ESC guideline assign these agents a Class I recommendation for the treatment of heart failure with reduced ejection fraction, irrespective of diabetes status. In heart failure with mildly reduced or preserved ejection fraction, SGLT2 inhibitors carry a Class IIa recommendation, supporting their use across the broader spectrum of left ventricular systolic function [31,32].

Guideline documents explicitly state that the indication for SGLT2 inhibitors in heart failure is independent of glycemic status. This recommendation is based on consistent reductions in the composite of cardiovascular death and heart failure hospitalization, as well as improvements in health-related quality of life observed across diabetic and nondiabetic subgroups in major trials [20,21,32].

Within guideline-directed medical therapy for heart failure with reduced ejection fraction, SGLT2 inhibitors are recognized as one of the four foundational pharmacologic classes, alongside beta-blockers, angiotensin receptor-neprilysin inhibitors (or renin-angiotensin system inhibitors), and mineralocorticoid receptor antagonists. Despite their formal endorsement, real-world utilization remains suboptimal, particularly among patients without diabetes, underscoring the persistent implementation gap between evidence and practice [33-35].

## Conclusions

SGLT2 inhibitors exert cardioprotective effects that extend beyond glycemic control through integrated metabolic, hemodynamic, and anti-inflammatory mechanisms. By modulating myocardial substrate utilization, improving cardiorenal interactions, and attenuating maladaptive remodeling, these agents function as disease-modifying therapies in heart failure irrespective of diabetes status. Robust evidence from large randomized trials demonstrates consistent reductions in heart failure hospitalization across reduced, mildly reduced, and preserved ejection fraction phenotypes, with mortality benefits most clearly established in reduced ejection fraction.

Supported by strong international guideline recommendations and a generally favorable safety profile in nondiabetic populations, SGLT2 inhibitors have become foundational components of contemporary heart failure management. Future efforts should prioritize early initiation, optimization of multidisciplinary implementation strategies, and continued investigation into mechanisms and long-term outcomes to maximize clinical benefit across diverse heart failure populations.
